# Theoretical Insight into Antioxidant Mechanism of Caffeic Acid Against Hydroperoxyl Radicals in Aqueous Medium at Different pH-Thermodynamic and Kinetic Aspects

**DOI:** 10.3390/ijms252312753

**Published:** 2024-11-27

**Authors:** Agnieszka Kowalska-Baron

**Affiliations:** Institute of Natural Products and Cosmetics, Faculty of Biotechnology and Food Sciences, Lodz University of Technology, Stefanowskiego 2/22, 90-537 Lodz, Poland; agnieszka.kowalska-baron@p.lodz.pl

**Keywords:** caffeic acid, antioxidant mechanism, HAT, SET, SPL-ET, SPL-HAT, RAF, free radical scavenging, DFT, pK_a_

## Abstract

In this study, the DFT/M062X/PCM method was applied to investigate thermodynamic and kinetic aspects of reactions involved in possible mechanisms of antioxidant activity of caffeic acid against HOO^●^ radicals in aqueous medium at different pH values. Kinetic parameters of the reactions involved in HAT (Hydrogen Atom Transfer), RAF (Radical Adduct Formation), and SET (Single Electron Transfer) mechanisms, including reaction energy barriers and bimolecular rate constants, were determined, and identification and characterization of stationary points along the reaction pathways within HAT and RAF mechanisms were performed. Inspection of geometrical parameters and spin densities of the radical products formed within HAT and RAF mechanisms revealed that they are stabilized by hydrogen bonding interactions and the odd electron originated through the reaction with the HOO^●^ radical is spread over the entire molecule, resulting in significant radical stabilization. Thermodynamic and kinetic data collected in this study indicated that increasing pH of the medium boosts the antioxidant activity of caffeic acid by reducing the energy required to generate radicals within the RAF and/or HAT mechanism and, at extremely high pH, where the trianionic form of caffeic acid is a dominant species, by the occurrence of an additional fast, diffusion-limited electron-related channel.

## 1. Introduction

Reactive oxygen species (ROS) belong to a group of free radicals and have an unpaired electron on the valence shell or in the excited state. Since they have excess energy, these species seek stability by accepting either a hydrogen atom or an electron. ROS are generated during essential metabolic processes in the human body (endogenous source of ROS) and they play a very important role in immune response and intracellular communication [1,2]. At low or moderate concentrations, ROS are substantial for human health [3]. However, ROS can also be derived exogenously from the exposure to harmful environmental factors, such as pollution, radiation, or xenobiotics [4]. The human body possesses several defense mechanisms to neutralize ROS. However, these may be insufficient and, as a consequence, uncontrolled accumulation of ROS may induce oxidative stress. In oxidative stress, ROS start a pathological chain reaction, leading to degeneration of biologically important systems such as DNA, proteins, and lipids, and to the development of many illnesses such as cancer, diabetes, atherosclerosis, and neurodegenerative diseases [5]. To protect biologically important targets from free radical-mediated damage and to maintain the balance between generated and neutralized ROS, external supplements of naturally occurring antioxidants are recommended [4].

**Figure 1:**
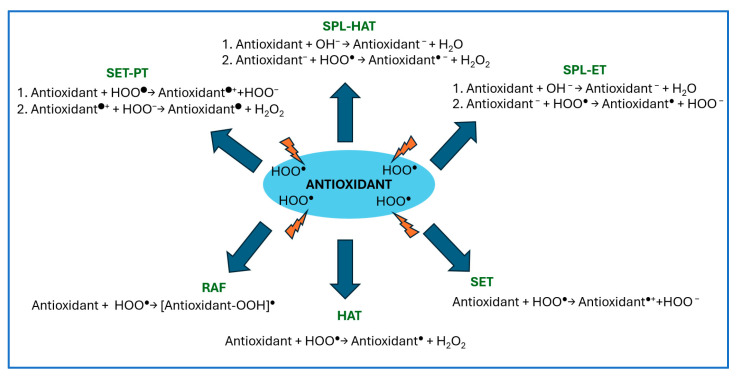
Schematic representation of mechanisms involved in free radical scavenging activity of type I antioxidants.

Antioxidants are compounds that can decrease the level of ROS and are categorized into three groups depending on their mechanism of action. The mechanism of action of type I antioxidants, called chain breakers, relies on their direct reactions with free radicals, leading to generation of more stable and less harmful species, which terminate chain reactions. These reactions usually involve hydrogen atom transfer (as a radical (HAT) or a proton transfer (PT)), or a single electron transfer (SET) that leads directly (HAT, SET) or through a stepwise mechanism through sequential proton loss SPL (SPL-HAT, SPL-ET) or SET-PT to the formation of more stable and less hazardous radical products. Such less harmful radicals may also be formed in the direct reaction between radicals and antioxidants involved in the radical adduct formation (RAF) mechanism [6] (Figure 1).

Examples of type II antioxidants are (1) metal chelators, which prevent Fenton reactions, thus limiting the formation of extremely reactive and harmful hydroxyl radicals; and (2) chromophores absorbing in the UV range. Compounds that take part in the repair of damaged biological targets of free radical are classified into the group of type III antioxidants. It should be noted that most antioxidants act via more than one of the above-mentioned mechanisms of action [6].

Caffeic acid (3,4-dihydroxycinnamic acid) is a polyphenolic secondary metabolite widely distributed in plants tissues and present in foods, including coffee drinks, blueberries, and apples, as well as in several medications based on propolis [2]. This compound was reported to exhibit antioxidant, immunomodulatory, antiviral, anti-HIV, anticarcinogenic, and ant-inflammatory activity [7,8]. In particular, the rate constant toward ROO^●^ radical scavenging by caffeic acid was experimentally determined to be 1.5 × 10^7^ M^−1^s^−1^ [9].

Caffeic acid can be classified into the group of type I and type II antioxidants. The results of the B3LYP,M052X/6-31+G(d)/C-PCM calculations performed by Leopoldini et al. [10] indicated that HAT and RAF are the most probable mechanisms for scavenging hydroxyl radicals by caffeic acid in the neutral and monoanionic form in the carboxylic functionality. The authors concluded that hydrogen abstraction more favorably occurs from the C_4_OH phenolic group (which is in the para position with respect to -C=C-COO^−^ (H^+^)), whereas radical addiction (within the RAF pathway) preferentially occurs in the C_4_ carbon atom (which is part of the aromatic ring). According to earlier authors, the SET mechanism was shown to be thermodynamically unfavorable in both polar (water) and nonpolar (benzene) media [10]. The studies of Redžepović et al. [11] confirmed that HAT and RAF mechanisms are two competitive antioxidant mechanisms of aqueous caffeic acid against hydroxyl radicals. The most stable radical formed within the HAT mechanism was the result of hydrogen abstraction from the phenolic group in the para position with respect to the chain consisting of two conjugated carbon atoms and the carboxyl group, whereas the most stable radical adduct was formed when hydroxyl radicals attacked one of the conjugated carbon atoms (-C=C-).

Redžepović et al. [11] ruled out SET-PT and SET pathways as an antioxidant mode of action of neutral form of caffeic acid in water and benzene (in these media caffeic acid is in its neutral form), but they suggested that a in polar basic environment, the SPL-ET mechanism is a very feasible option, as electron transfer from phenolate monoanions occurs with an extremely large rate with a diffusion control limit. More recent theoretical DFT studies by Purushothaman [12] on the thermodynamic and kinetic aspects of the mechanism of antioxidant activity of caffeic acid towards carbon-centered radicals, such as ^●^CH_2_OH and its isomeric form CH_3_O^●^, showed that the HAT mechanism is more feasible than RAF [12].

Previous studies showed that caffeic acid is able to effectively trap Fe(II) and Cu (II) [3,13,14] ions, thereby preventing Fenton- reactions and thus limiting the hydroxyl radical formation [3]. This mode of antioxidant action of caffeic acid is more efficient at higher pH values [13]. Moreover, at physiological pH, caffeic acid exhibits absorption in the UV range [14].

Though the antioxidant ability of caffeic acid in scavenging hydroxyl radicals has been extensively explored from both theoretical and experimental points of view, few studies have been devoted to determining their possible action as antioxidants against HOO^●^ radicals. These radicals may be formed by protonation of superoxide, an intermediate in biochemical reactions [15]. They are considered to be involved in tumor growth and they are also able to initialize lipid peroxidation. HOO^●^ radicals have longer half-life compared to hydroxyl radicals; the latter are so short-lived and reactive that they non-selectively attack almost any molecule in their vicinity at diffusion-limited rates, before an antioxidant can actually trap them [6,16]. It should be noted that although the deprotonated form of the HOO^●^ radical, that is O_2_^●−^, is typically present in physiological pH, it is an insufficiently reactive species to cause significant oxidative (oxidation) damage [6].

One of the studies on antioxidant mechanisms of selected polyphenols (including caffeic acid) against HOO^●^ radicals was published recently by Spiegel et al. [17], in which the authors considered three free radical scavenging mechanisms: HAT, SET-PT, and SPL-ET. The results of their B3LYP/6-311++G(d,p) calculations showed that HAT is a plausible mechanism of antioxidant activity of caffeic acid towards HOO^●^ radicals, whereas SET-PT was indicated by the authors as an unlikely mode of antioxidant action of caffeic acid in scavenging hydroperoxyl radicals.

In most theoretical studies on antioxidant mechanisms, methods based on Density Functional Theory have been applied. The choice of the appropriate exchange-correlation energy functional is a critical issue in DFT calculations. There is currently a trend towards the use of B3LYP, M052X, and its more recent version M062X [18], whereby the latter two functionals are more frequently used than B3LYP [6]. The popularity of M062X in overall antioxidant theoretical studies may be the result of its extensively documented good performance in prediction of (1) thermochemistry, energy barrier heights and noncovalent interactions [19,20]; and (2) reaction energies involving radicals [21], as well as rate constants of the radical reactions in aqueous medium [22]. Moreover, the studies of Spiegel et al. [23] indicated that M062X combined with the 6-311G(d,p) basis set is the best and most low-cost method to study antioxidative and antiradical actions of phytochemicals (with caffeic acid chosen as a reference structure).

However, the M062X functional is not without drawbacks and it should be used with care, since previous studies reported on the sensitivity of hybrid meta-generalized gradient approximation (m-GGA) functionals, including M062X, to the size of the integration grid and the basis set, which may lead to problems with geometry optimizations [24,25]. The grid errors exhibited by the M06 functionals arise from integration errors in the exchange component of the energy [25]. Moreover, the M062X performance strongly depends on the chemical systems [26].

Although M062X captures medium-range electron correlation, previous studies [27] showed that its application may lead to an inaccurate description of specific non-covalent interactions in biomolecules, due to the fact that in M062X the long-range electron correlation, which is important in this type of interaction, is neglected. The improved version of the original M06 suite of density functionals, a hybrid meta-generalized gradient-approximation functional revM06, which has improved accuracy for both main-group and transition-metal chemistry [28], has been developed.

In spite of the fact that the antioxidant activity of caffeic acid has been the subject of a few theoretical investigations, the mechanism of its antioxidant activity towards HOO^●^ radicals has not been fully clarified. In particular, so far there are no reports regarding the kinetic aspects of the free radical scavenging mechanism of caffeic acid against HOO^●^ radicals. Thermodynamic data are often insufficient in unravelling the free radical scavenging mechanism, since exergonic reactions can occur at different rates, either slow or fast, and on the other hand moderately endergonic processes (with ΔG < 10 kcal/mol), especially those involved in SET and RAF reactions, may still be relevant in overall antioxidant capacity [6,29].

The aim of this work is to contribute to the explanation of antioxidant activity of caffeic acid by examining all possible mechanisms of action of type I of the antioxidant (HAT, RAF, SET, SPL-ET, SET-PT, and SPL-HAT reaction pathways) in the presence of HOO^●^ hydroperoxyl radicals. For this purpose, the M062X/PCM method was applied to comprehensively investigate the electronic structure (indices related to Frontier Molecular Orbitals Theory were evaluated, and the most reactive sites for radical attack were indicated based on the calculated condensed Fukui functions [30]) and reactivity (both thermochemical intrinsic reactivity indices and Gibbs free energies of the reactions between caffeic acid and hydroperoxyl radicals involved in HAT, RAF, SET, SPL-ET, SPL-HAT, and SET-PT mechanisms were calculated) of caffeic acid towards HOO^●^ radicals in aqueous medium. Since the antioxidant activity of caffeic acid is affected by the pH of the environment [13], all possible acid–base species of caffeic acid were considered in this study and their acid–base equilibria were theoretically estimated with the use of different theoretical models with the aim of identifying the method which yields pK_a_ values that are more consistent with experimental data. In this study, the kinetic parameters of possible (from a thermodynamic point of view) pathways of inactivation of HOO^●^ radicals by caffeic acid were determined for the first time. Based on the obtained thermodynamic and kinetic data, the preferred pathways of the free radical scavenging capacity of caffeic acid towards HOO^●^ free radicals in media having different pH values were identified.

## 2. Results and Discussion

### 2.1. Theoretical Determination of Acid–Base Equilibria of Caffeic Acid (pK_a_ Values of Caffeic Acid)

**Figure 2:**
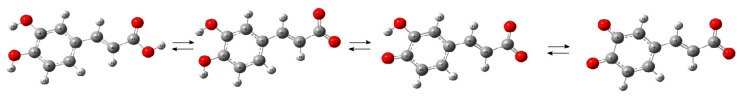
M062X/6-311+G(d,p)/PCM(water)-optimized forms (neutral and deprotonated) of caffeic acid. Atomic labels according to Gaussian 09.

Caffeic acid has free ionizable hydroxyl groups and is weakly acidic; therefore, depending on the pH value of the aqueous medium, four species of this compound are possible. M062X/6-311+G(d,p)/PCM(water)-optimized structures of neutral and deprotonated species of caffeic acid are shown in Figure 2.

Experimental pK_a_ values of caffeic acid were previously [13,31] reported to be 4.8, 8.6, and 11.2 (carboxylic group, p-hydroxy, and m-hydroxy group, respectively). As expected, the pK_a_ value of each subsequent deprotonation is increased since it is more difficult to detach a proton from the ion than from the neutral species. The calculated values of pK_a_ for caffeic acid with the use of the continuum cluster model with explicit water molecules, the isodesmic method, and the parameter-fitting method are gathered in Table 1.

As can be seen from Table 1, the cluster continuum method (with explicit water molecules) gives values of pK_a2_ quite close to experimental values, but the values of pK_a1_ and pK_a3_ are overestimated. This discrepancy may be related to the negligence of the basis set superposition error [33] in the method with explicit water molecules surrounding the corresponding ions.

Taking into account that variations of 0.7–2.4 units in the experimentally determined pK_a_ values of caffeic acid can also be found in the literature [14], the values for deprotonation of caffeic acid determined by the isodesmic method and the parameter-fitting method are quite consistent with the experimental values. Discrepancies between experimental and theoretically predicted values of pK_a1_ and pK_a2_ using isodesmic and parameter-fitting methods are less than 1.5 units.

The molar fractions of particular neutral and deprotonated species depend on their chemical structure and the pH of the medium. At physiological pH (7.4), the monoanionic form of caffeic acid prevails (93.84%), the fraction of the dianionic form is ~5.92%, while the fraction of the neutral and trianionic form is close to 0.

At high pH values (pH > 12), the trianionic form of caffeic acid prevails (98.44%; the dianionic form is present with a share of 1.56%, while neutral and monoanionic forms are practically absent). In trianionic forms of caffeic acid, all three hydroxyl groups are deprotonated, making hydrogen donation routes inaccessible, but the trianionic form of caffeic acid may still exhibit antioxidant activity through electron-related channels.

At pH < 4.8, neutral species of caffeic acid prevail. The presence of hydroxyl groups in these forms offers hydrogen-related channels in the free radical scavenging mechanism.

Thus, depending on the pH of the medium, four different forms of caffeic acid may exist. Since antioxidant activity of caffeic acid is affected by the pH of the environment [13], all possible acid–base species of caffeic acid will be considered in this study.

### 2.2. Prediction of Antioxidant Properties of Caffeic Acid Based on the Indices Related to Frontier Molecular Orbitals Theory

**Figure 3:**
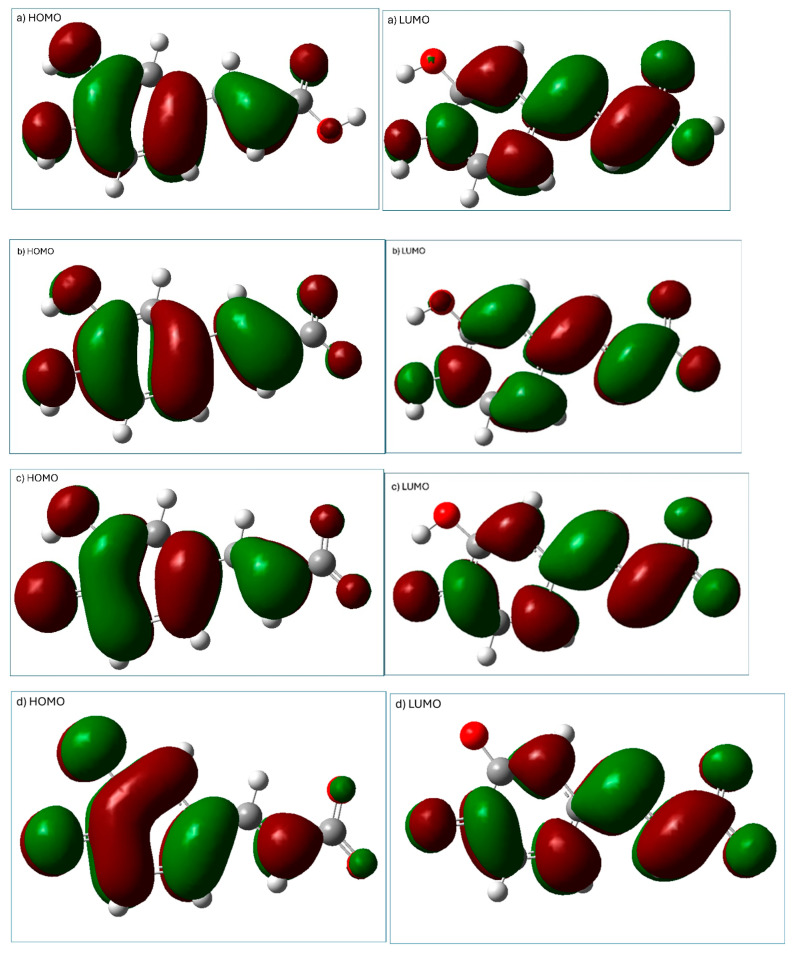
Visualization of HOMO and LUMO (calculated using M062X/6-31G(d,p)/PCM(water) method) of caffeic acid: (**a**) neutral form, (**b**) monoanion, (**c**) dianion, (**d**) trianion.

Energies and distribution of HOMO (the highest-occupied molecular orbital) and LUMO (the lowest-unoccupied molecular orbital) may provide preliminary information about antioxidant activity [6]. The hydroperoxyl radical is electrophilic; therefore, its interaction with antioxidants’ HOMO may be important [6]. Moreover, analysis of HOMO electronic distribution may be helpful in prediction of potential sites for radical attack. Representation of HOMO and LUMO orbitals of the four studied forms of caffeic acid (calculated using M062X/6-31G(d,p)/PCM(water) method) is shown in Figure 3.

The distribution of electronic density on HOMO and LUMO (Figure 3) indicates that for all the species, the aromatic ring is crucial in the electron-donating reaction with free radicals (HOMO distribution), whereas this ring also plays an important role in electron-accepting (LUMO distribution) free radical scavenging reactions.

Indices related to Frontier Molecular Orbitals Theory: electronegativity (χ), chemical potential (μ), global hardness (η), global softness (S), and electrophilicity (ω) were calculated and are gathered in Table 2. Electron-donating power (ω−) and electron-accepting power (ω+), were also calculated and are presented in Table 2.

From Table 2, it may be seen that deprotonation leads to the decrease in ionization potential (IP), electrophilicity (ω), electronegativity (χ), electro-donating power (ω−), and electron-accepting power (ω+). The lower the IP, χ, ω, ω− values, the more likely it is that the antioxidant molecule will act as an electron donor in interactions with other species. The lower the value of the electron-accepting power (ω+), global softness (S), and global hardness (η), the more unlikely it is that the antioxidant molecule will accept electrons in interactions with other species. The calculated indices (IP, ω, χ, ω−, ω+) suggest that each subsequent deprotonation increases the electron-donating properties of caffeic acid. The electron affinity (EA) value is positive for neutral species and negative for deprotonated species; EA decreases with each deprotonation.

It should be noted that in the applied approximations of orbitals eigenvalues as the ionization potential or electron affinity, electron correlation is ignored [6]. Moreover, orbital eigenvalues depend on the method and basis set employed in their calculations [6,23], which may lead to their inaccurate values and to invalid conclusions concerning the electrophilic character of the species [6].

### 2.3. Prediction of Radical Attack Site Based on the Condensed Fukui Functions

The condensed Fukui functions for radical attack $f_{A}^{0}$ calculated for an arbitrary atom A in a molecule allow the prediction of reactive sites in a more convenient way than the presentation of HOMO and LUMO orbitals, since $f_{A}^{0}$ values depend only on the atom’s electronegativity and electron density location [6]. Generally, the higher the $f_{A}^{0}$ value for a particular atom, the more prone that atom is to radical attack. The condensed Fukui functions for radical attack $f_{A}^{0}$ for C and O atoms of neutral and deprotonated species of caffeic acid are gathered in Table 3. It can be seen (Table 3) that among carbon atoms, the C_12_ of the conjugated carbon chain -C_11_=C_12_- is the most probable site for HOO^●^ radical attack, whereas from among oxygen atoms, the O_2_ of hydroxyl group, in the para position with respect to the conjugated carbon chain -C_11_=C_12_- with a carboxyl moiety, is the most reactive.

Table 3 shows relatively high values of $f_{A}^{0}$ for carbon atoms belonging to the aromatic ring. However, these values should not be considered as reliable, since previous studies [34] indicated that Fukui functions could not correctly predict the nucleophilic reaction sites for all aromatic molecules, and thus also sites for radical attack (since in the calculation of the condensed Fukui functions for radical attack, those for nucleophilic attack are used).

It should be noted that the values of atomic charges used to calculate condensed Fukui functions strongly depend on the population analysis method [6,35]. In this work, atomic charges were obtained from natural population analysis, which is reliant on the applied functional and basis set [36], as well as on the presence of the solvent [6,35].

Since the values of Fukui functions are related to the electron density of the frontier orbitals, they should be positive; meanwhile the negative value for C_13_ is noted for the monoanionic form of caffeic acid (see Table 3). Negative values of condensed Fukui functions were previously reported and explained as the result of small interatomic distances [6,37] and orbital expansion and contraction [38].

Although the condensed Fukui functions can be used to predict the possible radical attack sites in a very straightforward way, they do not provide information about the mechanism involved in the radical attack. However, this information can be gained from the calculated intrinsic thermochemical parameters.

### 2.4. Evaluation of Free Radical Scavenging Ability via Intrinsic Thermochemical Parameters

The intrinsic reactivity indices can give important preliminary data that will be helpful for evaluating the preferred antioxidant reaction pathways and showing the direction of further research. For example, bond dissociation enthalpy (BDE), adiabatic ionization potential (AIP), and proton affinity (PA) are considered as the primary indices of the HAT, SET, and SPL mechanisms, respectively. M062X/6-31G(d,p)/PCM(water) calculated values of AIP, electron transfer enthalpy ETE, BDE, PA, and proton dissociation enthalpy (PDE) are gathered in Table 4.

**Figure 4:**
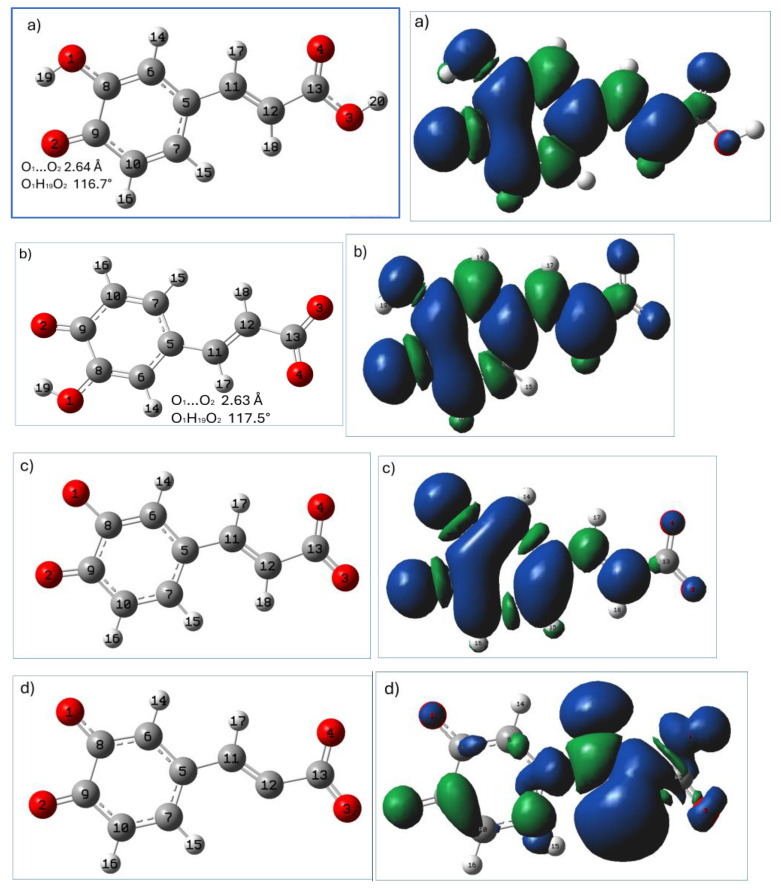
M062X/6-31G(d,p)/PCM(water) calculated geometries (**left** panel) of the most stable antioxidant radicals of (**a**) neutral, (**b**) monoanionic, (**c**) dianionic, and (**d**) trianionic species of caffeic acid formed in the HAT mechanism, together with their spin density distributions (**right** panel). Atomic labels according to Gaussian 09.

Radicals were formed through H detachment from the O_2_H_20_ group (neutral and monoanionic forms), O_1_H_19_ group (dianion), and C_12_H_18_ (trianion). Their geometries are presented in Figure 4. In calculations of indices involving the detachment of protons, the sequence of deprotonation was the same as in pK_a_ determination (carboxylic group, O_2_H_20_, O_1_H_19_).

AIP is characteristic of the SET mechanism and is defined as the minimum energy required to transfer an electron from the antioxidant to the free radical to form cationic radical species. The lower the IP value, the easier the electron is transferred and the higher the antioxidant activity via the SET mechanism. From Table 4, it may be noticed that AIP values decrease with deprotonation. Therefore, the importance of the SET pathway increases with the increase in pH of the medium [15]. Moreover, it can also be seen that the ETE values also decrease with the increase in ionization, indicating that deprotonation of hydroxyl groups favors electron-related channels.

The trianionic form of caffeic acid has the lowest value of adiabatic ionization potential (AIP); therefore, for this form, SET is the most probable free radical scavenging mechanism. The SET mechanism would proceed if the EA of a free radical is higher than the AIP of the antioxidant [6]. The EA of the free radical HOO^●^ is the enthalpy change accompanying the reaction R^●^ + e^−^ = R^−^ [15]. The M062X/6-31G(d,p)/PCM calculated value of EA for HOO^●^ is −47.52 kcal/mol. Thus, only for the trianionic form is the energy released by the free radical on gaining the electron sufficient to compensate for its AIP. Therefore, the trianion is expected to quench the radical by electron transfer. On the other hand, SET is unlikely for neutral, monoanionic, and dianionic forms of caffeic acid due to high adiabatic ionization potential.

From Table 4, it may be seen that each subsequent deprotonation of the hydroxyl group leads to a lower BDE value, which means that when increasing the pH value, it is easier to homolitically break the O-H bond. This suggests that the HAT pathway may be considered as a very likely mode of antioxidant action of caffeic acid, especially at that pH range where monoanionic and dianionic forms of the compound under study occur.

The stability of the antioxidant radical formed in the reactions involved in the antioxidant mechanism is a crucial requirement for an effective antioxidant. The graphical presentations of the most stable radicals of neutral, monoanionic, dianionic, and trianionic forms of caffeic acid formed i.a. within the HAT antioxidant mechanism (calculated at the M062X/6-31(d,p)/PCM(water) level) are shown in Figure 4, together with their spin density distributions.

The most stable radicals of neutral and monoanionic forms of caffeic acid arise from homolytic breakage of the O_2_H_20_ bond (in these structures O_2_H_20_ is in the para position with respect to the -C=C-COOH(COO^−^) group), while the remaining phenoxyl group provides stabilization via the intramolecular hydrogen bonding interaction (between O_2_ and H_19_O_1_). The better reactivity of H_20_O_2_ in the HAT mechanism is mainly due to the stability generated by this intramolecular hydrogen bond between the two phenolic groups. This hydrogen bond helps in stabilizing the electronic deficiency generated on the phenolic oxygen during the H abstraction reaction. In agreement with previously reported studies [11], radicals formed by the hydrogen atom subtraction from the O_1_H_19_ phenoxyl group of the neutral form of caffeic acid (presented in Supplementary Materials (Figure S1a)) are less stable (M062X/6-31G(d,p)/PCM predicted BDE for this radical assumed the value of 86.37 kcal/mol). The absence of an intramolecular hydrogen bonding interaction in this structure results in lower stability of the radical, and its spin density resulting from hydrogen atom removal is confined to the phenol ring (see Figure S1b).

In the spin density representation of the most stable radicals of neutral, monoanionic, and dianionic forms of caffeic acid (Figure 4a–c), the unpaired electron is delocalized over the entire planar molecule stabilizing the radical structure. The unpaired electron is mainly delocalized on the phenyl ring and vinyl bond (which is planar). The presence of the -CH=CH– bridge between phenyl and carboxyl groups favors resonance and conjugation effects.

In the trianionic form of caffeic acid there is no OH group, and the most stable radical in the HAT mechanism is that formed via H subtraction from C_12_ (the lowest BDE value for homolytic cleavage of the C-H group in the trianionic form was found at the C_12_H_18_ bond). However, this value is considerably higher than that for the other forms of caffeic acid, which suggest that the HAT mechanism is unlikely for the trianionic form of the studied compound. In the trianionic radical structure (Figure 4d, right panel), the unpaired electron is practically confined to the -CH=CH-COO^−^ moiety.

The proton-donating ability of caffeic acid is characterized by the calculated value of PA. For neutral and monoanionic forms of caffeic acid, PA values are lower than BDE values, suggesting that these forms are more prone to undergoing the SPL mechanism, which may be followed by the ET or HAT mechanism (Table 4). Moreover, the value of PA increases with the degree of ionization of hydroxyl groups, which is not surprising because each subsequent deprotonated species is less likely to detach another proton.

From Table 4, it may be seen that both PA and PDE values increase with the increase in ionization of hydroxyl groups, whereas the electron transfer enthalpy (ETE) decreases with the ionic character of the antioxidant, which is in line with the previously reported study [17].

These results indicate that with each subsequent deprotonation, routes that includes proton donation, e.g., SPL-ET, become more energetically demanding, but, on the other hand, electron-donating processes become more feasible.

The calculated intrinsic reactivity indices suggest that the possible antioxidant mechanism of the neutral form of caffeic acid includes both the HAT pathway or sequential deprotonation to anionic forms of this compound, and the subsequent HAT mechanism (this mechanism should be favored at the pH range where monoanionic and dianionic forms of caffeic acid are present), while for deprotonated forms of caffeic acid (the trianionic form), the SET mechanism should be considered as a possible free radical scavenging mode of action.

### 2.5. Possible Pathways of HOO^●^ Free Radical Scavenging by Caffeic Acid

Since antioxidant action depends not only on the antioxidant itself, but also on radicals with which it interacts, the Gibbs free energy associated with the reactions with HOO^●^ radicals related to HAT, RAF, SET, SET-PT, SPL-ET, and SPL-HAT mechanisms (Table 5) can give more important data than intrinsic reactivity indices alone. The HOO^●^ radical is less reactive than the non-selective HO^●^ radical, which instantly reacts at a diffusion-limited rate with almost any molecule in its proximity, before an antioxidant is able to intercept it. The HOO^●^ radical has a long enough half-life and is considered as a main contributor to oxidative damage [6].

When considering the RAF mechanism, two reactive sites, at C_11_ and C_12_ of the -C=C-COO^-^(H^+^) conjugated chain for radical addition, were taken into account. The carbons of the double bond were also previously indicated as the most favorable sites in caffeic acid for HO^●^ attack [11]. However, another study [10] showed that these radicals also (and even preferably) form radical adducts (within the RAF mechanism) with the carbon of the phenolic group in the para position with respect to the carboxyl group. This indicates that hydroxyl radicals HO^●^ in RAF reaction are non-selective and very reactive.

The calculated ΔG of the reaction involved in the RAF pathway showed that formation of radical adducts at C_11_ is not spontaneous and thermodynamically favorable (ΔG > 0 than kcal/mol, see Table S2, Supplementary Materials). Moreover, with each deprotonation of caffeic acid, the RAF reaction to carbon C_11_ is more endergonic. The M062X/6-31G(d,p)/PCM-optimized geometries of the radicals formed after HOO^●^ attack at C_11_ are shown in Figure S2, left panel (Supplementary Materials), whereas the selected geometrical parameters of these radicals are gathered in Table S1 (Supplementary Materials). In the final RAF products formed as the result of radical addition to C_11_, the conjugation between the -CH=CH-COO^−^ (H^+^) group and the aromatic group is lost (see the values of C_5_C_11_ and C_5_C_11_C_12_). Moreover, the addition of a radical to C_11_ causes a notable deviation of the carbon chain skeleton from the aromatic ring plane (see the value of C_6_C_5_C_11_C_12_ torsion angle), which leads to disruption of electron delocalization between the benzene ring and acyclic chain. As a consequence, the resultant radicals are less stable (as compared to radicals formed after HOO^●^ addition to C_12_) since the unpaired electron is mainly confined to the carboxylic (carboxylate) group and carbon chain (see spin distributions of these radicals, Figure S2, right panel). The results of kinetic calculations indicated that the energy barrier of the HOO^●^ addition to C_11_ of the neutral form of caffeic acid is high (the calculated activation energy ΔG_a_^#^ assumes the value of 97.417 kJ/mol and the bimolecular rate constant is close to 0 (k_bim_ 5.317 × 10^−5^ M^−1^s^−1^)). A graphical presentation of the stationary points (reactant complex (RC), transition state (TS), product complex (PC)) encountered during the reaction involved in RAF (C_11_) is shown in Figure S3, Supplementary Materials). Thus, the obtained data indicate that the addition of HOO^●^ radicals to C_11_ of caffeic acid is unlikely from both thermodynamic and kinetic points of view.

On the other hand, the results of calculations indicated that addition of hydroperoxyl radicals to C_12_ is moderately endergonic, but each subsequent deprotonation of caffeic acid favors formation of radical adducts at C_12_ (for monoanionic, dianionic, and trianionic forms of caffeic acid, the RAF(C_12_) reaction is exergonic; see Table 5).

**Figure 5:**
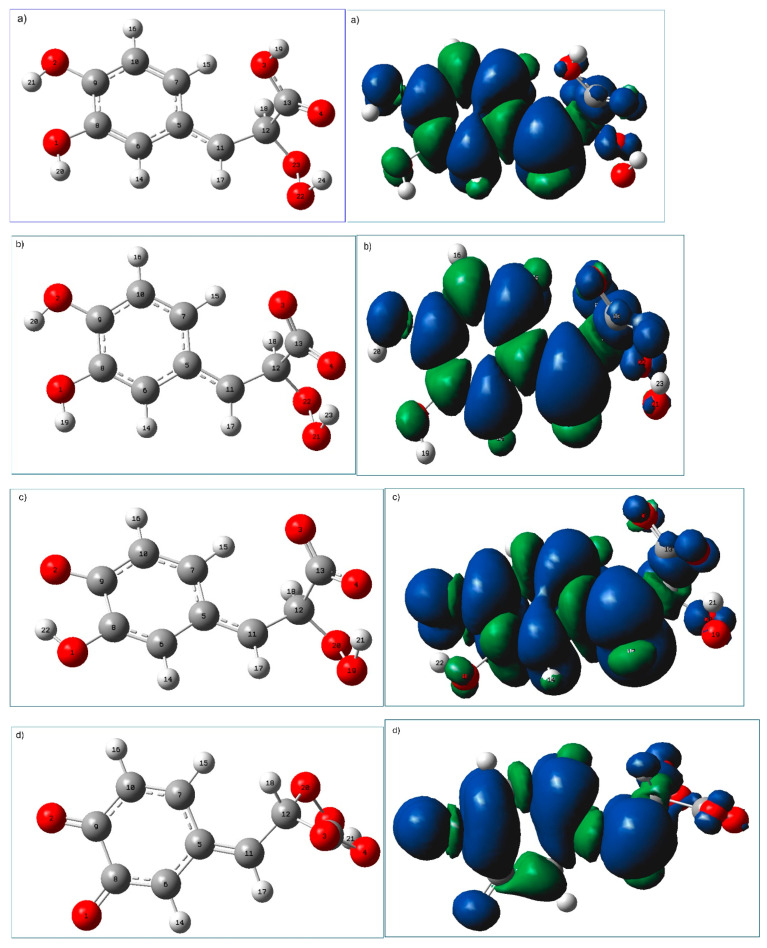
M062X/6-31G(d,p)/PCM(water) calculated geometries (**left** panel) of the most stable radical adducts of (**a**) neutral, (**b**) monoanionic, (**c**) dianionic, and (**d**) trianionic species of caffeic acid formed in the radical adduct formation (RAF-C_12_) mechanism together with their spin density distribution (**right** panel). Atomic labels according to Gaussian 09.

The geometries of radical adducts formed as the result of HOO^●^ addition to C_12_ of neutral and deprotonated forms of caffeic acid are presented in Figure 5, together with their spin density distributions. Inspection of the geometrical parameters of these adducts (gathered in Table 6) reveals that the products of hydroperoxyl radical addition to neutral and deprotonated forms of caffeic acid generally show similar geometrical features: formation of radical adducts involves a particular rearrangement of the carboxylic or carboxylate group (which in the final product is perpendicular to the aromatic ring) to facilitate the hydrogen bonding interaction of its carbonyl oxygen with the attached radical (see Table 6; geometrical parameters of these hydrogen-bonded interactions are marked with bold font). In the RAF products, the C_5_C_11_ bond, which is in the nearby surroundings of the phenol ring, is shorter than in the parent molecules, and C_5_C_11_C_12_ is 125°, while the C_11_C_12_C_13_ bond angle is around 110°. Moreover, in the RAF (C_12_) adducts, the acyclic carbon chain is almost linear with an aromatic ring (see the values of C_6_C_5_C_11_C_12_ in Table 6), which favors resonance and delocalization effects. As the result, the unpaired electron originated through the reaction with the free radicals has the possibility to be spread over the entire molecule, resulting in a significant radical stabilization (see spin density distribution in Figure 5, right panel).

From Table 5, it may be seen that the reaction involved in the HAT mechanism is exergonic with neutral species of caffeic acid when H is subtracted from O_2_. On the other hand, the results of the calculation showed that the HAT reaction involving less-stable radicals (formed after H subtraction from the H_19_O_1_ phenoxyl group of the neutral form of caffeic acid; see Figure S1a, Supplementary Materials) is endergonic (the calculated ΔG for the HAT reaction was 1.374 kcal/mol). Deprotonation of COOH group makes hydrogen subtraction from hydroxyl group more feasible (easier), as each subsequent deprotonation of the hydroxyl group leads to the decrease in the Gibbs free energy of the reaction involved in the HAT mechanism (see Table 5). It should be noted that in the case of monoanionic and dianionic forms of caffeic acid, the Gibbs free energy of HAT is equivalent to (is identical to) the second step of the SPL-HAT mechanism (see Table 5). In other words, because the deprotonation that initiates the SPL-HAT is already included indirectly by investigating various deprotonated species of caffeic acid, for deprotonated species, this process can be described in terms of HAT.

From Table 5, it may be noticed that for the trianionic form of caffeic acid, the reaction of hydrogen atom subtraction from C_12_ is endergonic; therefore, at high pH values, the HAT mechanism leading to the formation of this kind of radical of caffeic acid is unlikely.

The Gibbs energy of the reaction involved in the SET mechanism decreases with the ionic character of the antioxidant, and becomes negative for the trianionic form of caffeic acid, which suggests that, in a highly basic medium, the SET mechanism may be considered a feasible mode of free radical scavenging activity. For neutral, monoanionic, and dianionic forms of caffeic acid, the SET mechanism is not thermodynamically favorable (as the formation of radical cations is a highly endergonic process). It should be noted that in the case of ionic forms of caffeic acid, the Gibbs free energy of SET is the same as the second step of the SPL-ET mechanism (see Table 5).

Both SPL-HAT and SPL-ET mechanisms are initiated by proton dissociation, the proclivity of which is controlled by a medium pH and acid–base equilibrium. The results of calculations showed that in the presence of a base (OH^−^), the proton dissociation is highly exergonic. Each subsequent dissociation is more energetically demanding (less exergonic) due to the increasing negative charge of the initial structure, which makes proton detachment more difficult.

Since (1) the first step of the SET-PT mechanism is endergonic for neutral, monoanionic, and dianionic forms of caffeic acid, and (2) the second step of this mechanism is endergonic for the trianionic form of the studied compound, SET-PT may be considered as an unfavorable pathway of free radical scavenging by caffeic acid. This is in line (consistent) with previously reported studies [11,17].

Reactions with free radicals are irreversible and subject to kinetic control [6]. Thermodynamics is used to predict the direction of spontaneous chemical reactions of stage I reactions at equilibrium, but it says nothing about how quickly the reaction approaches equilibrium. An exergonic reaction can occur at either fast or slow rates [6].

It has been recently evidenced that the Gibbs free energy of the reaction involved in the HAT mechanism is proportional to the activation energy of this reaction, and hence the reaction rate of this mechanism [6]. Moderately endergonic reactions (with delta G less than 10 kcal/mol), particularly within RAF and SET mechanisms, may still contribute to antioxidant activity [6]. The reaction between the neutral form of caffeic acid and HOO^●^ radicals leading to the formation of radical adducts is not exergonic, but lower than the recommended threshold of 10 kcal/mol, implying that this route may still contribute to antioxidant activity. The Gibbs free energies of the other endergonic reactions are significantly higher than the mentioned threshold. These reactions are thermodynamically unfavorable and kinetic calculations will not be performed for them.

For SET reactions, thermochemical and kinetic data may show opposing trends [6,29]. Therefore, the assumptions deduced from the thermodynamic consideration of the RAF mechanism for the neutral form of caffeic acid, as well as the SET antioxidant mechanism of the trianionic form of caffeic acid, need to be completed with kinetic investigations.

### 2.6. Kinetics of Reactions Involved in HAT, SET and RAF Mechanism

In contrast to the SET mechanism, hydrogen atom abstraction and radical addition reactions involved in HAT and RAF mechanisms, respectively, proceed through TS. Therefore, stationary points along the reaction pathways within HAT and RAF mechanisms have to be carefully investigated.

**Figure 6:**
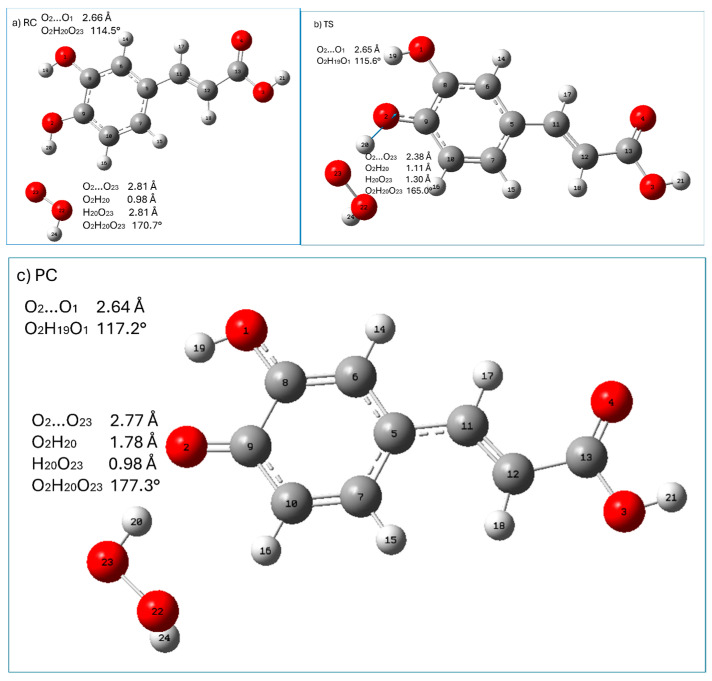
Graphical presentation of the stationary points ((**a**) reactant complex (RC), (**b**) transition state (TS), and (**c**) product complex (PC)) encountered along the HAT reaction pathway of caffeic acid (neutral form) with hydroperoxyl radicals. Displacement vectors of imaginary frequency at 1555.320*i* are shown as blue arrows. Atomic labels according to Gaussian 09.

**Figure 7:**
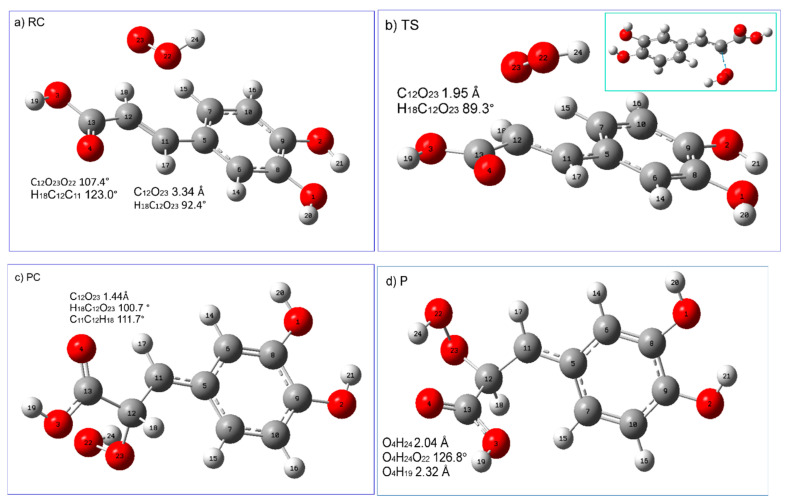
Graphical presentation of the stationary points ((**a**) reactant complex (RC), (**b**) transition state (TS), (**c**) product complex (PC), and (**d**) product (P)) encountered along the RAF (C_12_) reaction pathway of caffeic acid (neutral form) with hydroperoxyl radical. Displacement vectors of imaginary frequency at 454.67*i* are shown as blue arrows. Atomic labels according to Gaussian 09.

Graphical presentation of the stationary points (reactant complex (RC), transition state (TS), and product complex (PC)) along the HAT reaction pathway of caffeic acid (neutral form) with hydroperoxyl radicals are presented in Figure 6, whereas those encountered in the RAF mechanism are shown in Figure 7. The relative enthalpies and Gibbs free energies of the stationary points encountered along the reaction pathways within HAT and RAF mechanisms with respect to the reactants are gathered in Table 7.

All HAT stationary points (RC, TS, PC) are characterized by a significant stabilization resulting from the presence of intramolecular hydrogen bonding interaction between the O_1_H_19_ group and O_2_ oxygen atoms. Additional stabilization of the RC and TS intermolecular systems arise from the hydrogen bonding interaction between HOO^●^ radicals and the H_20_O_2_ group, whereas in PC, the H_2_O_2_ molecule forms a hydrogen bond with the O_2_ oxygen atom. Geometrical parameters of the latter indicate that these interactions are strong (the distance between electronegative atoms taking part in theses interactions is less than 3 Å and they are nearly linear; see Figure 6).

The hydrogen abstraction reaction within the HAF mechanism passes through a TS (imaginary frequency 1555.320*i* cm^−1^ corresponding to the stretching of the O_2_H_20_ bond) in which the H_20_ atom attached to O_2_ is transferred to the radical (it is indicated by the change in the H_20_O_23_ distance from 2.81 Å in RC, 1.30 Å in TS, and 0.98 Å in PC; see Figure 6).

From Table 7, it may be noticed that in the case of the HAT mechanism all stationary points have lower enthalpy than the reactants. The reaction involved in the HAT mechanism (leading to the formation of PC and products) is exothermic and exergonic. The free Gibbs energy of RC and TS is higher than that of the reagents. This is most probably due to the decrease in entropy related to the reduction in the degrees of freedom in the system caused by directional and ordered hydrogen bonding interactions. Both relative enthalpy and free energy increase from RC up to TS and then decrease down to product complex. When going from PC to separated products, the Gibbs energy continues to decrease, which can be related to the positive entropy change of separated products: hydrogen peroxide (H_2_O_2_) molecules and antioxidant radicals (the reduced amount of order and the increased degrees of freedom of separated products).

In the RAF reaction pathway, the HOO^●^ radical approaches almost perpendicularly (O_23_C_12_H_18_ is 92.4°) the caffeic acid (at the C_12_O_23_ distance of 3.24 Å; see Figure 7a), weakly interacting with π electrons of C_12_ of the double-bonded carbon chain (-C=C-), (C_11_C_12_ is 1.35 Å, H_18_C_11_C_12_ is 123.0°). The H atom of the radical is directed towards the aromatic ring interacting with its π electrons. This leads to stabilization of the resulting reactant complex, in which the conjugated -C=C- COOH chain is planar (C_5_C_11_C_12_C_13_ is 180°) and colinear with the phenyl ring. The hydroperoxyl radical is attached to C_12_ through the TS state, the nature of which was confirmed by the presence of one imaginary frequency at 454.67*i* cm^−1^ corresponding to the stretching of the C_12_-O_23_ bond. In TS, C_12_ retains geometrical features of carbon sp^2^ hybridization (H_18_C_12_C_11_ is 120.6°; C_11_C_12_ is 1.35 Å) and the conjugated carbon chain is nearly planar (C_5_C_11_C_12_C_13_ is 171.3°). In TS, the distance between C_12_O_23_ is shortened to 1.95 Å, and the angle H_18_C_12_O_23_ is 89.3°. TS evolves into PC, in which the C_12_O_23_ bond is completely formed (C_12_O_23_ 1.44 Å; H_18_C_12_O_23_ 100.7°; H_18_C_12_C_11_ 111.7°; H_18_C_12_C_13_ 106.6°). After the transition of TS, conjugation is lost, although atoms partially retained planarity (C_5_C_11_C_12_C_13_ 169.6°). It should be noticed that in PC there is no hydrogen bonding interaction of the hydroxyl group of the attached radical with the carboxyl oxygen of the COOH group, whereas this intramolecular hydrogen bonding interaction appears in the final RAF product. Formation of this hydrogen bond was facilitated by specific rearrangement of the -COOH group (C_5_C_11_C_12_C_13_ 90.8°; see Table 6), leading to the product with the radical OH group pointing towards the carbonyl oxygen of the -COOH group of caffeic acid. Geometrical parameters of this bond (O_4_O_22_ distance assumes the value 2.73 Å; O_4_H_24_O_22_ is 126.8°; Table 6) suggest that it is quite strong and contributes to the stabilization of the resultant radical adduct.

In the RAF mechanism, RC and TS have higher enthalpy than the reactants (Table 7). The enthalpy increases when going from RC to TS, and then decreases, and the overall reaction leading to formation of PC and P is exothermic. The Gibbs free energy increases up to TS (which is related to the decrease in entropy caused by the reduced degree of freedom in the molecular system), and then decreases when the PC and final product are formed, but the overall reaction is moderately (<10 kcal/mol) endergonic. This may indicate that the decrease in entropy associated with the formation of intramolecular hydrogen bonds incorporating the radical hydroxyl group and carbonyl oxygen atom of the carboxylic group in the final RAF product makes a greater contribution to the reaction Gibbs energy as compared to the enthalpy term (the decrease in entropy has a greater value than the enthalpy of the reaction, and the reaction Gibbs free energy is positive).

Kinetic parameters of the exergonic reactions involved in the SET and HAT antioxidant mechanisms (for trianionic and neutral forms of caffeic acid, respectively) towards HOO^●^ are gathered in Table 8, together with the kinetic data for the moderately endergonic addition of hydroperoxyl radicals to the neutral form (at C_12_) of the compound under study (within the RAF pathway).

From Table 8, it may be seen that the energy barrier for the RAF reaction is almost three times higher than that for the HAT reaction. The bimolecular rate constant of the HAT reaction is 1.48 × 10^9^ M^−1^s^−1^ at 298 K, meaning that it is very fast, whereas the reaction involved in the RAF mechanism is evidently slower (the calculated value of the bimolecular rate constant of radical adduct formation within RAF at 298 K is six orders of magnitude lower compared to that corresponding to HAT). Since it is generally considered [6] that the reaction Gibbs free energy is proportional to the rate constant of the reactions involved in HAT and RAF mechanisms, that is, the more exergonic the reaction, the faster the reaction, it is reasonable to expect that in the medium at physiological pH, where monoanionic forms of caffeic acid prevail, HAT and RAF reactions will proceed with faster rate constants. However, further research that aims at determining the kinetic parameters of the reactions involved in HAT and RAF mechanisms incorporating deprotonated forms of caffeic acid is needed and welcome.

**Figure 8:**
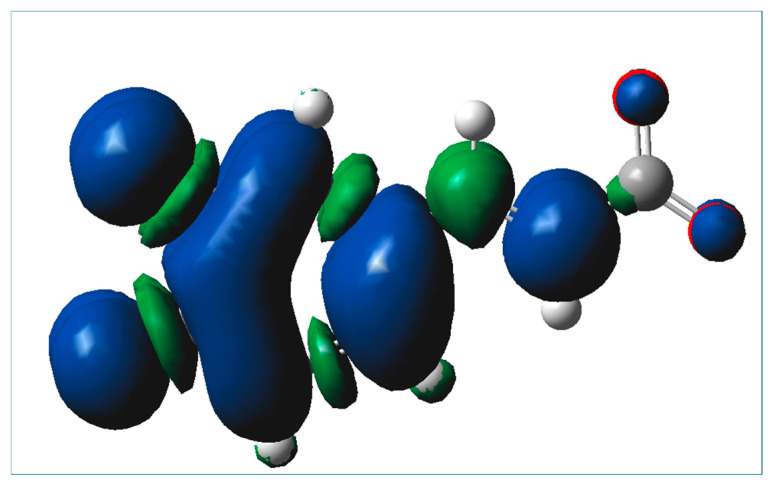
Spin density of the resultant radical formed in the SET mechanism involving the trianionic form of caffeic acid.

From the determined kinetic parameters of the reaction involved in the SET pathway (Table 8) that is expected to occur in extremely basic aqueous environments, where the trianionic form of caffeic acid prevails, it is evident that this exergonic reaction is characterized by a low energy barrier, and the determined bimolecular rate constant is very high, above the diffusion limit (3.19 × 10^11^ M^−1^s^−1^). From Table 8, it may be noted that the absolute value of the Gibbs free energy of the reaction involved in the SET mechanism is higher than the reorganization energy (Table 8), indicating that the reaction is in the inverted region of the Marcus parabola, where the reaction barrier increases as ΔG decreases [6,39]. However, the energy barrier of the reaction is very low and the rate of the reaction is limited only by the diffusion. The spin density of the resultant radical formed in the SET mechanism involving the trianionic form of caffeic acid is presented in Figure 8, from which it may be seen that the radical is a planar species, as the parent molecule and the unpaired electron are mainly spread over the aromatic ring and the CH=CH- chain, resulting in significant radical stabilization.

## 3. Computational Methods

### 3.1. Electronic and Geometrical Structure of Caffeic Acid

Geometry optimization and subsequent frequency calculations of the molecular structures of H_2_O_2_ and caffeic acid (in neutral, monoanionic, dianionic, and trianionic forms) and corresponding radicals were conducted at the M062X/6-31G(d,p)/PCM(water) level of theory. This level of theory is a compromise between theoretical outcomes and computational resource uptake. For closed-shell systems, restricted computations were performed, while for open-shell systems (radicals), the unrestricted calculation schemes were applied. Analysis of the calculated frequencies was performed to confirm the nature of the obtained stationary points; no imaginary frequencies were obtained for local minima, whereas transition states were characterized by only one imaginary frequency. All calculations were performed with the use of Gaussian 09W [40] and the GaussView 5.0 suite of programs [41].

### 3.2. Indices Related to Frontier Molecular Orbitals Theory

Indices related to Frontier Molecular Orbitals Theory for caffeic acid were calculated using the M062X/6-31G(d,p)/PCM(water) method. The ionization potential (IP) and electron affinity (EA) of the isolated species were evaluated according to Janak’s theorem [42,43].

IP=−E(HOMO)EA=−E(LUMO)

where E(HOMO)-M062X/6-31G(d,p)/PCM(water) is the calculated energy of the highest occupied orbital, and E(LUMO)-M062X/6-31G(d,p)/PCM(water) is the calculated energy of the lowest unoccupied orbital.

Having these values, descriptions of indices related to Frontier Molecular Orbitals Theory—electronegativity (χ), chemical potential (μ), global hardness (η), global softness (S), and electrophilicity (ω)—were calculated from the following formulas [44,45,46]:

$$\chi =\frac{IP+EA}{2}$$

$$\mu =-\chi =\frac{-IP+EA}{2}$$

$$\eta =\frac{IP-EA}{2}$$

$$S=\frac{\eta }{2}$$

$$\omega =\frac{{µ}^{2}}{2\eta }$$

Electro-donating power (ω−) and electron-accepting power (ω+) were calculated from the following formulas [6]:

$$\omega -=\frac{{\left(3IP+EA\right)}^{2}}{16\left(IP-EA\right)}$$

$$\omega +=\frac{{\left(IP+EA\right)}^{2}}{16\left(IP-EA\right)}$$

### 3.3. Prediction of Radical Attack Site Based on Condensed Fukui Functions

Electron population analysis (at the M062X/6-31G(d,p)/PCM level of theory) was performed with the use of natural bonding orbitals (NBOs). The obtained atomic charge values of (N − 1) and (N + 1) systems were used to calculate condensed Fukui indices for each atom A of the caffeic acid molecule (in both neutral and deprotonated forms) according to the following equation:

$$f_{A}^{0}=\frac{\left(q_{N-1}^{A}-q_{N+1}^{A}\right)}{2}$$

where $f_{A}^{0}$ denotes the Fukui index for free radical attack of atom A; and $q_{N-1}^{A}$ and $q_{N+1}^{A}$ denote the atomic charge of atom A in the molecule with (N − 1) and (N + 1) electrons, respectively, obtained vertically from the optimized ground-state geometry of a molecule with N electrons.

### 3.4. Intrinsic Reactivity Indices

Bond dissociation enthalpy (BDE), defined as the amount of energy necessary for homolytic breakage of the bond (the lower the BDE value, the more stable the radical formed) related to the following reaction:

Antioxidant → Antioxidant^●^ + H^●^

was calculated from the following equation:

BDE → H(Antioxidant^●^) + H(H^●^) −H(Antioxidant)

where H(Antioxidant), H(Antioxidant^●^), and H(H^●^) are the M062X/6-31G(d,p)/PCM(water) calculated values of enthalpy of the antioxidant, antioxidant radical, and hydrogen radical, respectively.

Adiabatic ionization potential (AIP), defined as the ability to donate an electron related to the reaction

Antioxidant → Antioxidant^●+^ + e^−^

was calculated from the following equation:

AIP → H(Antioxidant^●+^) + H(e^−^) −H(Antioxidant)

where H(e^−^) is the enthalpy of the solvated (water) electron (−98.8 kJ/mol) [47] and H(Antioxidant^●+^) is the M062X/6-31G(d,p)/PCM(water) calculated enthalpy of the cation radical.

Proton affinity (PA), defined as the amount of energy required to break the bond related to the following reaction:

Antioxidant→ Antioxidant^−^ + H^+^

was calculated using the following equation:

PA = H(Antioxidant^−^) + H(H^+^)−H(Antioxidant)

where H(Antioxidant^−^) is the M062X/6-31G(d,p)/PCM(water) calculated enthalpy of the respective deprotonated anion, and H(H^+^) is the enthalpy of the solvated (water) proton (−1052.7 kJ/mol) [47].

Proton dissociation enthalpy (PDE), defined as the amount of energy needed to break the bond during the following reaction:

Antioxidant^●+^ → Antioxidant^●^ + H^+^

was calculated from the following equation:

PDE = H(Antioxidant)^●^ + H(H^+^)−H(Antioxidant^●+^)

Electron transfer enthalpy (ETE), which characterizes the anion propensity for electron donation, was calculated from the following equation:

ETE = H(Antioxidant^●^) + H(e^−^)−H(Antioxidant^−^)

and related to the following reaction:

Antioxidant^−^→Antioxidant^●^ + e^−^

The enthalpies of solvated proton and electron were taken from [47].

### 3.5. Thermochemistry of HOO^●^ Free Radical Scavenging Reaction Pathways

There are six possible mechanisms through which antioxidant compounds (of type I chain breakers) exhibit their free radical scavenging action: HAT (hydrogen atom transfer), RAF (radical adduct formation), SET (single electron transfer), SET-PT (sequential electron transfer–proton transfer), SPL-ET (sequential proton loss–electron transfer), and SPL-HAT (sequential proton loss–hydrogen atom transfer) [6].

HAT is a one-step process described by the following reaction:

Antioxidant + HOO^●^ → Antioxidant^●^ + H_2_O_2_

The Gibbs free energy of the reaction is used to describe this mechanism.

This mechanism can be described using the BDE of the active OH/CH bonds of the antioxidant.

RAF is a process where the [Antioxidant-OOH]^●^ adduct is in the following reaction:

Antioxidant + HOO^●^ → [Antioxidant−OOH]^●^

The Gibbs free energy of the reaction is used to describe this mechanism.

SET involves an electron transfer from the neutral or anionic state of the antioxidant to the radical. This process can be described by the Gibbs free energy of the following reaction:

Antioxidant + HOO^●^ → Antioxidant^●+^ + HOO^−^

The SET-PT mechanism occurs in two steps: first a radical cation is formed by electron transfer from an antioxidant to radical, and then it deprotonates:

1. Antioxidant + HOO^●^→ Antioxidant^●+^+HOO^−^;

2. Antioxidant^●+^ + HOO^−^→ Antioxidant^●^ + H_2_O_2_.

The Gibbs free energy of the above-mentioned reactions are used to describe this mechanism.

In the SPL-ET mechanism in an aqueous medium, at first in the presence of a conjugated base OH^-^, the antioxidant is deprotonated, and then electron transfer occurs from the deprotonated antioxidant to the radical:

1. Antioxidant + OH^−^→ Antioxidant^-^ + H_2_O;

2. Antioxidant^−^ + HOO^●^ → Antioxidant^●^ + HOO^−^.

The Gibbs free energy of the above-mentioned reactions are used to describe this mechanism.

The SPL-HAT mechanism is similar to that of SPL-ET, except that instead of an electron, a hydrogen atom is transferred in the second step:

1. Antioxidant + OH^−^→ Antioxidant^−^ + H_2_O;

2. Antioxidant^−^ + HOO^●^ → Antioxidant^●−^ + H_2_O_2_.

### 3.6. Rate Constant for Single Electron Transfer (SET) Reaction

In the SET reaction, there is no transition state (TS) between reactants and products. The energy barrier for electron transfer (SET mechanism) was calculated on the basis of Marcus theory [48,49], according to the following equation:

$${\Delta G}_{ET}=\frac{\lambda }{4}{\left(1+\frac{{\Delta G}_{ET}^{0}}{\lambda }\right)}^{2}$$

where λ—nuclear reorganization energy, calculated as the difference between the ΔE (the vertical energy between reactants and products of the reaction via SET mechanism) and the adiabatic free energy of the reaction ${\Delta G}_{ET}^{0}$:

$$\lambda =\Delta E-{\Delta G}_{ET}^{0}$$

The bimolecular rate constant for the SET reaction (k_bim_) was calculated in the framework of the 1 M standard state [50] using the Eyring equation [51]:

$$k_{bim}=\frac{k_{B}T}{c^{‡}h}exp\left(\frac{-{\Delta G}_{ET}}{RT}\right)$$

where $c^{‡}\;$= 1 M molar concentration, k_B_—Boltzmann constant, h—Planck constant, R—gas constant, T—absolute temperature.

For bimolecular rate constants close to the diffusion limit, apparent rate constants k_app_ were calculated based on Collins–Kimball theory [52,53]:

$$k_{app}=\frac{k_{D}k_{bim}}{k_{D}+k_{bim}}$$

where k_D_ is the rate constant for an irreversible bimolecular diffusion-controlled reaction calculated following Smoluchowski [54]:

$$k_{D}=4\pi R_{AB}D_{AB}N_{A}$$

where N_A_ is the Avogadro number; $R_{AB}$ is the distance at which the reaction takes place, calculated as the sum of the reactants’ radii; and $D_{AB}$ is the mutual diffusion coefficient of the antioxidant A and radical B calculated using the Stokes–Einstein approach [55,56] as the sum of the corresponding diffusion coefficients:

$$D_{A}=\frac{k_{B}T}{{6\pi \eta R}_{A}}$$

$$D_{B}=\frac{k_{B}T}{{6\pi \eta R}_{B}}$$

where η is the viscosity of water (0.8905·0.001 Pa·s [57]).

### 3.7. Rate Constants for HAT and RAF Reactions

In HAT and RAF mechanisms, TS is encountered along the reaction pathways. It is assumed that in HAT and RAF mechanisms, the respective two reactants (antioxidant and hydroperoxyl radical) form the reactant complex RC and the process is considered as a unimolecular reaction of the RC [11]. Then, bimolecular rate constants for the reactions involved in HAT and RAF pathways were calculated in the framework of the 1 M standard state using the Eyring equation [51].

$$k_{bim}=\frac{k_{B}T}{c^{‡}h}exp\left(\frac{-{\Delta G}_{\hat{a}}^{\#}}{RT}\right)$$

where $c^{‡}$ = 1 M molar concentration, k_B_—Boltzmann constant, h—Planck constant, R—gas constant, T—absolute temperature, ${\Delta G}_{\hat{a}}^{\#}$—the free activation energy.

In order to find and optimize transition state structures in HAT and RAF reaction pathways, the method implemented by Schlegel and coworkers [58,59] was applied (requested by the QST3 option in Gaussian 09). Then, for the identified TS, the intrinsic reaction coordinate (IRC) was constructed starting from the respective TS geometry and going downhill to both the reactant and the product channels to ensure that the obtained TS corresponds to the reactions involved in HAT and RAF mechanisms.

### 3.8. Deprotonation Constants

The pK_a_ values for three-step caffeic acid deprotonation were calculated using three methods: the continuum cluster model with explicit water molecules, the relative (isodesmic) method, and the parameter-fitting method [6,60,61,62]. The free energy of solvated species was calculated with the use of the M062X,B3LYP/6-311+G(d,p)/PCM(water) method.

**Continuum cluster model with explicit water molecules**

In the continuum cluster model [60], acid deprotonates into ions which are solvated by water molecule(s) according to the following reactions:

(1) H_3_A+ OH^−^(3 H_2_O) → H_2_A^−^(H_2_O) + 3 H_2_O;

(2) H_2_A^−^(H_2_O) + OH^−^(3 H_2_O) → HA^2−^(2 H_2_O) + 3 H_2_O;

(3) HA^2−^(2 H_2_O) + OH^−^(3 H_2_O) → A^3−^(3 H_2_O) + 3 H_2_O.

Water is used as a co-reactant in pK_a_ calculations because it simplifies the modeling of protons and overcomes the computational difficulties associated with accurately estimating proton solvation energy [60].

In this model, the pK_a_ values corresponding to the three deprotonation steps were calculated using the following equation:

$${pK}_{a}=\frac{{\Delta G}_{s}}{2.303\cdot RT}+14+3log\left[H_{2}O\right]$$

where ${\Delta G}_{s}$ is the free energy of the appropriate deprotonation reaction, calculated as the difference between free energy of solvated products and reactants [J/mol]; R is the gas constant [J/molK], T is the standard temperature [K], [$H_{2}O$] = 55.55 mol/L (standard state of liquid water).

**Isodesmic method**

The isodesmic method relies on the proton exchange equilibrium between the caffeic acid and the conjugated base of the reference acid (Ref), according to the following reactions:

(1) H_3_A + H_2_A^−^(Ref)→ H_2_A^−^ + H_3_A(Ref);

(2) H_2_A^-^+ HA^2−^(Ref)→ HA^2−^ + H_2_A^−^(Ref);

(3) HA^2−^ + A^3−^(Ref)→ A^3−^ + HA^2−^(Ref).

In pK_a_, determination of caffeic acid, 3,4-dihydroxybenzoic acid was taken as the reference compound (pK_a_ values of 3,4-dihydroxybenzoic acid (pK_a1_ = 4.48; pK_a2_ = 8.83; pK_a3_ = 12.6) taken from [32].

The pK_a_ values corresponding to all the deprotonation steps (pK_a1_, pK_a2_, pK_a3_) of caffeic acid were calculated from the following equation:

$${pK}_{a}=\frac{{\Delta G}_{s}^{*}}{2.303\cdot RT}+pK_{a}\left(Ref\right)$$

where ${\Delta G}_{s}^{*}$ is the free energy of the appropriate deprotonation reaction [6], calculated as the difference between the free energy of solvated products and reactants [J/mol], R is the gas constant [J/molK], T is the standard temperature [K].

**Parameter-fitting method**

Using the parameter-fitting method [61,62], the pK_a_ values corresponding to each of the deprotonation steps (pK_a1_, pK_a2_, pK_a3_) of caffeic acid were calculated from the following equation:

$$pK=m\Delta G_{prot/deprot}+C$$

where $\Delta G_{prot/deprot}$ denotes the difference in the Gibbs free energy between the conjugated base and corresponding acid (expressed in [kcal/mol]), and m and C are empirical parameters taken from [61].

The experimental values of pK_a_s of caffeic acid [13] were used to calculate molar fractions of neutral and deprotonated species of caffeic acid as a function of pH.

## 4. Conclusions

In this work, thermodynamic and kinetic aspects of the reactions involved in possible mechanisms of HOO^●^ free radical scavenging activity of caffeic acid in an aqueous medium at different pH values were systematically investigated. At low pH values, where the neutral form of caffeic acid prevails, the HAT mechanism is the most probable; the reaction involved in this pathway is exothermic and exergonic with the determined bimolecular rate constant equal to 1.48 × 10^9^ M^−1^s^−1^. The reaction of radical addition to C_12_ of the neutral form of caffeic acid, involved in the RAF mechanism, is less exothermic and moderately endergonic. Kinetic calculations showed that this reaction occurs with the bimolecular rate constant that is six orders of magnitude lower compared to that corresponding to HAT, which indicates that the RAF mechanism makes less contribution to the antioxidant activity of caffeic acid in the pH range where neutral forms of caffeic acid prevail. Inspection of geometrical parameters and spin densities of the radical products formed within HAT and RAF mechanisms revealed that they are stabilized by intramolecular hydrogen bonding interactions, and the odd electron originating through the reaction with HOO^●^ radicals is spread over the entire molecule, resulting in significant radical stabilization.

Increasing pH of the medium leads to deprotonation of hydroxyl groups and the appearance of monoanionic and dianionic forms of caffeic acid in which RAF and HAF pathways are more feasible, since the reactions involved in these pathways become more exergonic. It is also expected that at higher pH values, where monoanionic and dianionic forms of caffeic acid are present, these reactions will proceed with higher rates. In an extremely basic medium where the trianionic form of caffeic acid prevails, the antioxidant action of the studied compound is mainly due to SET and RAF mechanisms. Kinetic investigations showed that the rate of the reaction involved in the SET mechanism is extremely fast and limited solely by the diffusion.

Thermodynamic data presented here showed that the first step of the SET-PT mechanism is endergonic for neutral, monoanionic, and dianionic forms of caffeic acid, and the second step of this mechanism is endergonic for the trianionic form of the studied compound. This allows the exclusion of the SET-PT mechanism in contributing to the free radical scavenging ability of caffeic acid against HOO^●^ radicals.

The results presented in this study confirmed the experimental findings, which showed that increasing the pH of the medium boosts the antioxidant activity of caffeic acid by reducing the energy required to generate radicals within the RAF or HAT mechanism, and by the occurrence of an additional fast, diffusion-limited electron-related channel at high pH values where the trianionic form of caffeic acid prevails.

## Figures and Tables

**Table 1:** M062X/6-311+G(d,p)/PCM(water) calculated values of pK_a_ for caffeic acid with the use of different theoretical approaches together with the experimental values taken from the literature [13,14]. Values calculated with the use of B3LYP/6-311+G(d,p)/PCM(water) are given in brackets.

pK_a_	Cluster-Continuum Model	Isodesmic Method *	Parameters Fitting Method	Literature Experimental Values
pK_a1_	5.9 (6.7)	5.7 (5.8)	6.2 (6.2)	4.8 [13]; 3.6–4.49 [14]
pK_a2_	8.0 (8.4)	7.6 (8.7)	9.2 (8.9)	8.6 [13]; 8.6–9.3 [14]
pK_a3_	20.6 (21.8)	9.6 (9.0)	16.3 (15.3)	11.2 [13]; 10.3–12.7 [14]

* pK_a_ values of reference compound (3,4-dihydroxybenzoic acid; pK_a1_ = 4.48; pK_a2_ = 8.83; pK_a3_ = 12.6) taken from [32].

**Table 2:** M062X/6-31G(d,p)/PCM(water) calculated indices related to Frontier Molecular Orbitals Theory.

Caffeic Acid	IP [kcal/mol]	EA [kcal/mol]	Χ [kcal/mol]	μ [kcal/mol]	η [kcal/mol]	S [kcal/mol	ω [kcal/mol]	ω− [kcal/mol]	ω+ [kcal/mol]
neutral	164.02	18.99	91.51	−91.51	72.52	36.26	57.74	112.56	21.05
monoanion	154.06	−4.22	74.92	−74.92	79.14	39.57	35.46	82.81	7.89
dianion	117.23	−20.02	48.60	−48.60	68.63	34.31	17.21	50.09	1.49
trianion	82.40	−31.36	25.52	−25.52	56.88	28.44	5.73	25.60	0.07

**Table 3:** The condensed Fukui indices $f_{A}^{0}$ for O and C atoms in neutral and deprotonated species of caffeic acid.

Atom	Neutral	Monoanion	Dianion	Trianion
O_1_	0.043	0.039	0.042	0.115
O_2_	0.076	0.055	0.151	0.124
O_3_	0.041	0.049	0.050	0.050
O_4_	0.070	0.046	0.042	0.041
C_5_	0.066	0.084	0.080	0.026
C_6_	0.025	0.032	0.019	0.067
C_7_	0.079	0.099	0.064	0.081
C_8_	0.050	0.042	0.056	0.018
C_9_	0.105	0.126	0.051	0.047
C_10_	0.013	0.005	0.057	0.050
C_11_	0.078	0.055	0.059	0.083
C_12_	0.132	0.184	0.156	0.125
C_13_	0.042	−0.002	0.011	0.021

Atomic labels according to Gaussian 09—see Figure 2.

**Table 4:** M062X/6-31G(d,p)/PCM(water) calculated values of adiabatic IP (AIP), ETE, BDE, PA, and PDE.

Caffeic Acid	AIP [kcal/mol]	ETE [kcal/mol]	BDE [kcal/mol]	PA [kcal/mol]	PDE [kcal/mol]
neutral	112.37	74.71	79.02	44.81	1.63
monoanion	102.23	66.15	76.20	44.84	8.94
dianion	66.14	30.80	73.51	77.68	42.34
trianion	30.80	-	108.55	-	112.72

AIP—adiabatic ionization potential, ETE—electron transfer enthalpy, BDE—bond dissociation energy, PA—proton affinity. PDE—proton dissociation enthalpy.

**Table 5:** M062X/6-31G(d,p)/PCM(water) Gibbs free energy [kcal/mol] at 298.15 of the reactions involved in the HAT, SET, SET-PT, SPL-ET, and SPL-HAT mechanisms towards HOO^●^ radicals.

Caffeic Acid	RAF ΔG [kcal/mol]	HAT ΔG [kcal/mol]	SET ΔG [kcal/mol]	SET-PT ΔG [kcal/mol]	SPL-ET ΔG [kcal/mol]	SPL-HAT ΔG [kcal/mol]
neutral	2.51 (C_12_)	−5.16 (O_2_)	64.92	(1) 64.92 (2) −70.08 (O_2_)	(1) −47.64 * (2) 54.86	(1) −47.64 (2) −8.43
monoanion	−9.40 (C_12_)	−8.43 (O_2_)	54.86	(1) 54.86 (2) −63.29 (O_2_)	(1) −46.39 (2) 18.23	(1) −46.39 (2) −11.78 (O_2_)
dianion	−10.77 (C_12_)	−11.78 (O_1_)	18.23	(1) 18.23 (2) −30.00 (O_1_)	(1) −14.53 (2) −17.33	(1) −14.53 (2) 22.88 (C_12_)
trianion	−15.93 (C_12_)	22.88 (C_12_)	−17.33	(1) −17.33 (2) 39.86	-	-

* (monoanion).

**Table 6:** M062X/6-31G(d,p)/PCM(water) calculated geometrical parameters of the most stable radical adducts of different species of caffeic acid with HOO^●^ radicals.

Neutral		Monoanion		Dianionic		Trianionic	
Bond Length	[Å]	Bond Length	[Å]	Bond Length	[Å]	Bond Length	[Å]
C_5_C_11_	1.41	C_5_C_11_	1.41	C_5_C_11_	1.40	C_5_C_11_	1.40
C_11_C_12_	1.50	C_11_C_12_	1.49	C_11_C_12_	1.50	C_11_C_12_	1.48
C_12_C_13_	1.53	C_12_C_13_	1.56	C_12_C_13_	1.56	C_12_C_13_	1.54
C_13_O_3_	1.33	C_13_O_3_	1.24	C_13_O_3_	1.24	C_13_O_3_	1.24
C_13_O_4_	1.21	C_13_O_4_	1.27	C_13_O_4_	1.27	C_13_O_4_	1.24
C_12_O_23_	1.42	C_12_O_22_	1.43	C_12_O_20_	1.43	C_12_O_20_	1.46
O_23_O_22_	1.42	O_22_O_21_	1.43	O_20_O_19_	1.44	O_20_O_19_	1.44
O_22_H_24_	0.97	O_21_H_23_	1.01	O_19_H_21_	1.02	O_19_H_21_	1.01
O_4_H_24_	2.04	O_4_H_23_	1.58	**O_1_O_2_**	**2.58**	**O_19_O_4_**	**2.52**
**O_22_O_4_**	**2.73**	**O_21_O_4_**	**2.51**	**O_19_O_4_**	**2.50**	**bond angle**	**[°]**
**bond angle**	**[°]**	**bond angle**	**[°]**	**bond angle**	**[°]**	C_5_C_11_C_12_	124.9
C_5_C_11_C_12_	125.0	C_5_C_11_C_12_	125.3	C_5_C_11_C_12_	125.3	C_11_C_12_C_13_	110.8
C_11_C_12_C_13_	111.7	C_11_C_12_C_13_	112.1	C_11_C_12_C_13_	112.6	C_11_C_12_O_20_	116.2
C_11_C_12_O_23_	113.2	C_11_C_12_O_22_	111.2	C_11_C_12_O_20_	111.8	O_20_O_19_H_21_	97.1
O_23_O_22_H_24_	100.5	O_22_O_21_H_23_	96.9	O_20_O_19_H_21_	96.8	**O_19_H_21_O_4_**	**154.0**
**O_22_H_24_O_4_**	**126.8**	**O_21_H_23_O_4_**	**151.3**	**O_19_H_21_O_4_**	**153.0**	**dihedral**	**[°]**
**dihedral**	**[°]**	**dihedral**	**[°]**	**O_1_H_22_O_2_**	**125.5**	C_5_C_11_C_12_C_13_	155.2
C_5_C_11_C_12_C_13_	90.8	C_5_C_11_C_12_C_13_	83.1	**dihedral**	**[°]**	O_19_O_20_C_12_C_13_	65.1
O_22_O_23_C_12_C_13_	67.6	O_21_O_22_C_12_C_13_	61.7	C_5_C_11_C_12_C_13_	86.0	H_21_O_19_O_20_C_12_	−50.6
H_24_O_22_O_23_C_12_	−80.3	H_23_O_21_O_22_C_12_	−56.6	O_19_O_20_C_12_C_13_	62.5	O_4_C_13_C_12_O_20_	−37.7
O_4_C_13_C_12_O_23_	−11.8	O_4_C_13_C_12_O_22_	−23.0	H_21_O_19_O_20_C_12_	−54.3	C_6_C_5_C_11_C_12_	178.5
C_6_C_5_C_11_C_12_	179.0	C_6_C_5_C_11_C_12_	174.8	O_4_C_13_C_12_O_20_	−28.1		
				C_6_C_5_C_11_C_12_	176.0		

Geometrical parameters of hydrogen bonded interactions are marked with bold font; atomic labels according to Gaussian 09.

**Table 7:** The calculated relative enthalpy H [kJ/mol] and relative free energy values G [kJ/mol] of the stationary points along the reaction coordinate in HAT and RAF mechanisms. The values were calculated with respect to those for isolated reagents.

	RC		TS		PC		Products	
	H [kJ/mol]	G [kJ/mol]	H [kJ/mol]	G [kJ/mol]	H [kJ/mol]	G [kJ/mol]	H [kJ/mol]	G [kJ/mol]
HAT	−19.985	15.540	−5.535	36.221	−62.765	−24.895	−34.961	−35.591
RAF	2.657	28.513	37.030	83.614	−9.255	39.574	−16.016	35.471

**Table 8:** Kinetic parameters of the reactions involved in the SET, HAT, and RAF antioxidant mechanisms of the trianionic form (SET) and neutral form (HAT, RAF) of caffeic acid towards HOO^●^ radicals.

	ΔG_r_ [kJ/mol]	ΔE [kJ/mol]	λ [kJ/mol]	ΔG_a_^#^ [kJ/mol]	k_bim_ [M^−1^s^−1^]	k_D_ [M^−1^s^−1^]	k_app_ [M^−1^s^−1^]
SET	−72.56	63.22	135.75	7.36	3.19 × 10^11^	1.31 × 10^7^	1.31 × 10^7^
HAT	−23.49	-	-	20.68	1.48 × 10^9^	-	-
RAF	10.49	-	-	55.10	1.38 × 10^3^	-	-

## Data Availability

Data are contained within the article.

## Supplementary Materials

The following supporting information can be downloaded at: https://www.mdpi.com/article/10.3390/ijms252312753/s1.
