# Supplementary material for: Theoretical Insight into Antioxidant Mechanism of Caffeic Acid Against Hydroperoxyl Radicals in Aqueous Medium at Different pH-Thermodynamic and Kinetic Aspects
Source: Int J Mol Sci. 2024 Nov 27;25(23):12753. doi: 10.3390/ijms252312753 (PMC11641784; doi:10.3390/ijms252312753)
Supplement: Supplementary file 1 [file ijms-25-12753-s001.zip › ijms-3333951-supplementary.pdf]

SUPPLEMENTARY MATERIALS

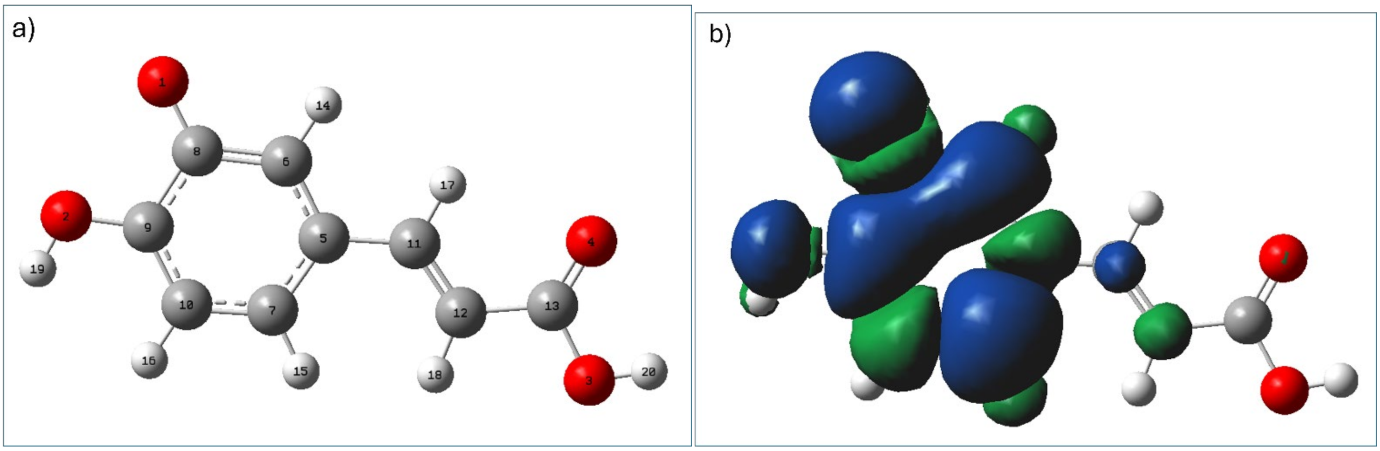

Figure S1. M062X/6-31G(d,p)/PCM optimized geometry of radical obtained via H detachment from O1 of neutral form of caffeic acid (**a**) together with its spin density distribution (**b**).

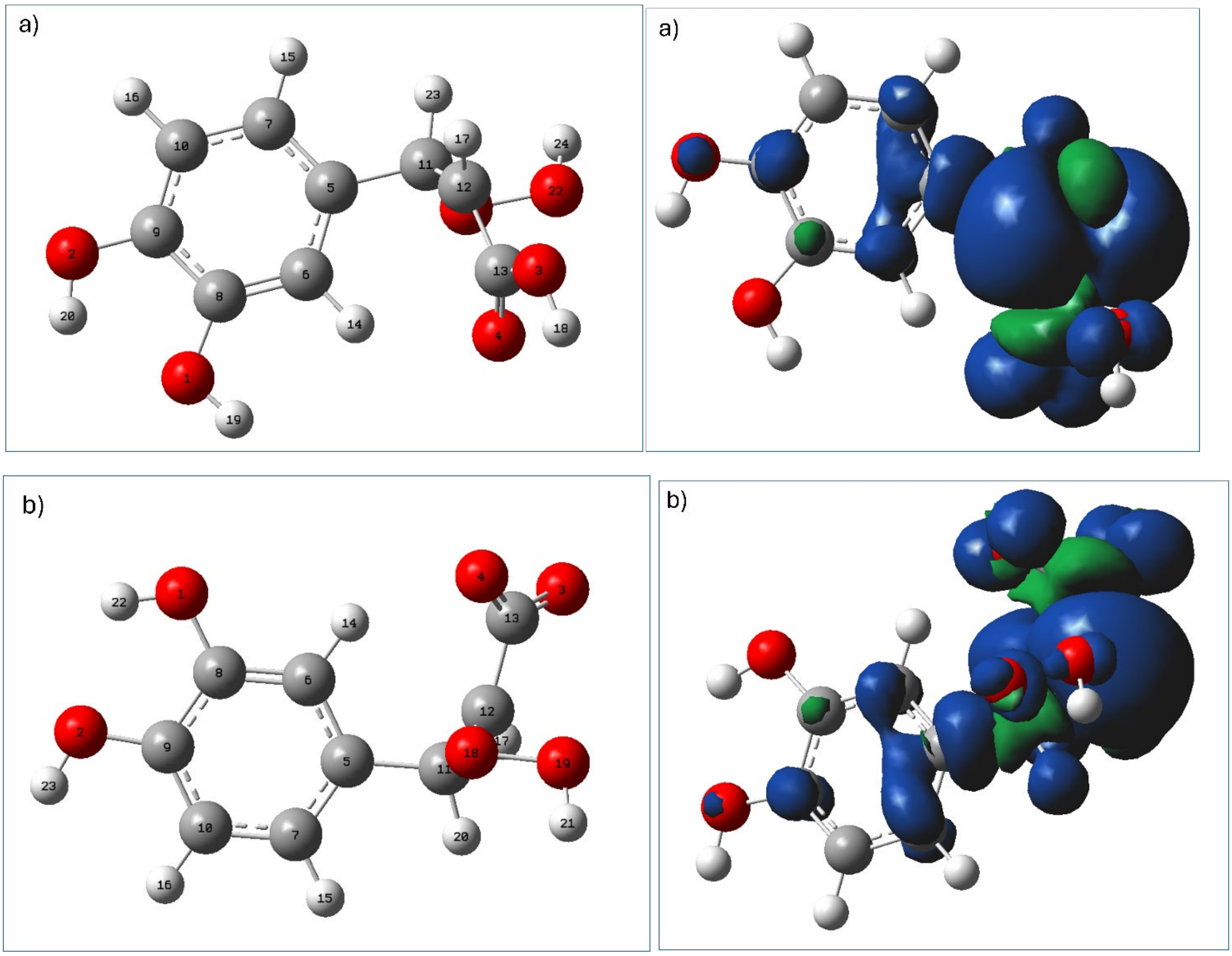

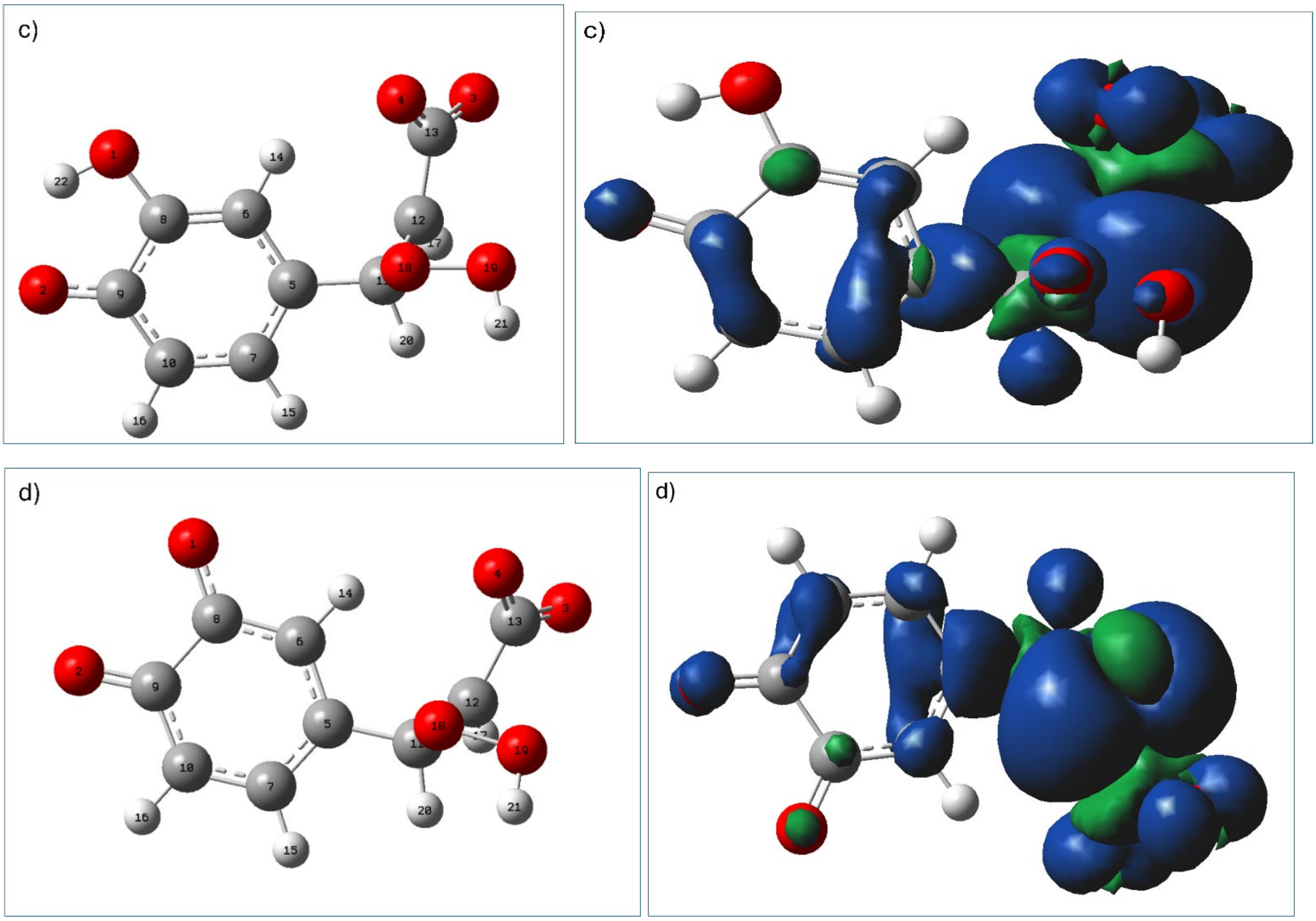

Figure S2. M062X/6-31G(d,p)/PCM optimized geometries of radicals obtained through HOO• addition to C_{11} of (**a**–**d**) neutral and deprotonated forms of caffeic acid.

Table S1. M062X/6-31G(d,p)/PCM optimized geometrical parameters of radicals obtained via HOO• addition to C_{11} of neutral and deprotonated forms of caffeic acid.

Neutral		Monoanionic		Dianionic		Trianionic	
**Bond Length**	**[Å]**	**Bond Length**	**[Å]**	**Bond Length**	**[Å]**	**Bond Length**	**[Å]**
C_5C_{11}	1.51	C_5C_{11}	1.51	C_5C_{11}	1.51	C_5C_{11}	1.51
$C_{11}C_{12}$	1.50	$C_{11}C_{12}$	1.50	$C_{11}C_{12}$	1.50	$C_{11}C_{12}$	1.50
$C_{12}C_{13}$	1.45	$C_{12}C_{13}$	1.50	$C_{12}C_{13}$	1.50	$C_{12}C_{13}$	1.49
$C_{13}O_3$	1.35	$C_{13}O_3$	1.26	$C_{13}O_3$	1.26	$C_{13}O_3$	1.27
$C_{13}O_4$	1.22	$C_{13}O_4$	1.26	$C_{13}O_4$	1.26	$C_{13}O_4$	1.26
$C_{11}O_{24}$	1.42	$C_{11}O_{18}$	1.43	$C_{11}O_{18}$	1.44	$C_{11}O_{18}$	1.44
$O_{21}O_{22}$	1.42	$O_{18}O_{19}$	1.42	$O_{18}O_{19}$	1.43	$O_{18}O_{19}$	1.43
$O_{22}H_{24}$	0.97	$O_{19}H_{21}$	0.97	$O_{19}H_{21}$	0.97	$O_{19}H_{21}$	0.97
O_4H_{24}	4.07	O_4H_{21}	4.33	O_1O_2	2.56	$O_{19}O_4$	3.30
$O_{22}O_4$	3.11	$O_{18}O_4$	2.98	$O_{19}O_4$	3.38	**bond angle**	**[°]**
**bond angle**	**[°]**	**bond angle**	**[°]**	**bond angle**	**[°]**	$C_5C_{11}C_{12}$	114.5
$C_5C_{11}C_{12}$	111.9	$C_5C_{11}C_{12}$	113.9	$C_5C_{11}C_{12}$	114.1	$C_{11}C_{12}C_{13}$	129.6

$C_{11}C_{12}C_{13}$	124.1	$C_{11}C_{12}C_{13}$	127.4	$C_{11}C_{12}C_{13}$	128.3	$O_{18}C_{11}C_{12}$	113.7
$C_{12}C_{11}O_{21}$	113.0	$C_{12}C_{11}O_{18}$	113.9	$C_{12}C_{11}O_{18}$	113.7	$O_{18}O_{19}H_{21}$	101.5
$O_{21}O_{22}H_{24}$	101.7	$O_{18}O_{19}H_{21}$	101.6	$O_{18}O_{19}H_{21}$	101.5	$O_{4}O_{19}O_{21}$	162.2
$O_{4}O_{22}H_{24}$	168.3	$O_{4}O_{19}H_{21}$	161.7	$O_{4}O_{19}H_{21}$	162.1	**dihedral**	**[°]**
**dihedral**	**[°]**	**dihedral**	**[°]**	$O_{1}H_{22}O_{2}$	126.7	$C_{5}C_{11}C_{12}C_{13}$	90.4
$C_{5}C_{11}C_{12}C_{12}$	87.7	$C_{5}C_{11}C_{12}C_{13}$	84.6	**dihedral**	**[°]**	$O_{19}O_{18}C_{11}C_{12}$	-57.7
$O_{22}O_{21}C_{11}C_{13}$	-55.3	$O_{19}O_{18}C_{11}C_{12}$	-57.8	$C_{5}C_{11}C_{12}C_{13}$	85.3	$H_{24}O_{19}O_{18}C_{11}$	-89.3
$H_{24}O_{22}O_{21}C_{11}$	-94.0	$C_{11}O_{18}O_{19}H_{21}$	-94.6	$O_{19}O_{18}C_{11}C_{12}$	-57.2	$O_{18}C_{11}C_{12}C_{13}$	-32.6
$O_{21}C_{11}C_{12}C_{13}$	-31.7	$C_{13}C_{12}C_{11}O_{18}$	-35.6	$C_{11}O_{18}O_{19}H_{21}$	-93.3		
$C_{6}C_{5}C_{11}C_{12}$	-62.8	$C_{6}C_{5}C_{11}C_{12}$	-53.4	$C_{13}C_{12}C_{11}O_{18}$	-36.3	$C_{6}C_{5}C_{11}C_{12}$	-51.2
				$C_{6}C_{5}C_{11}C_{12}$	-52.1		

Table S2. M062X/6-31G(d,p)/PCM calculated Gibbs free energy of $HOO^{\bullet}$ radical addition to C_{11} of neutral and deprotonated forms of caffeic acid.

**Caffeic Acid**	**ΔG [kcal/mol]**
neutral	7.547
monoanion	8.044
dianion	8.946
trianion	10.420

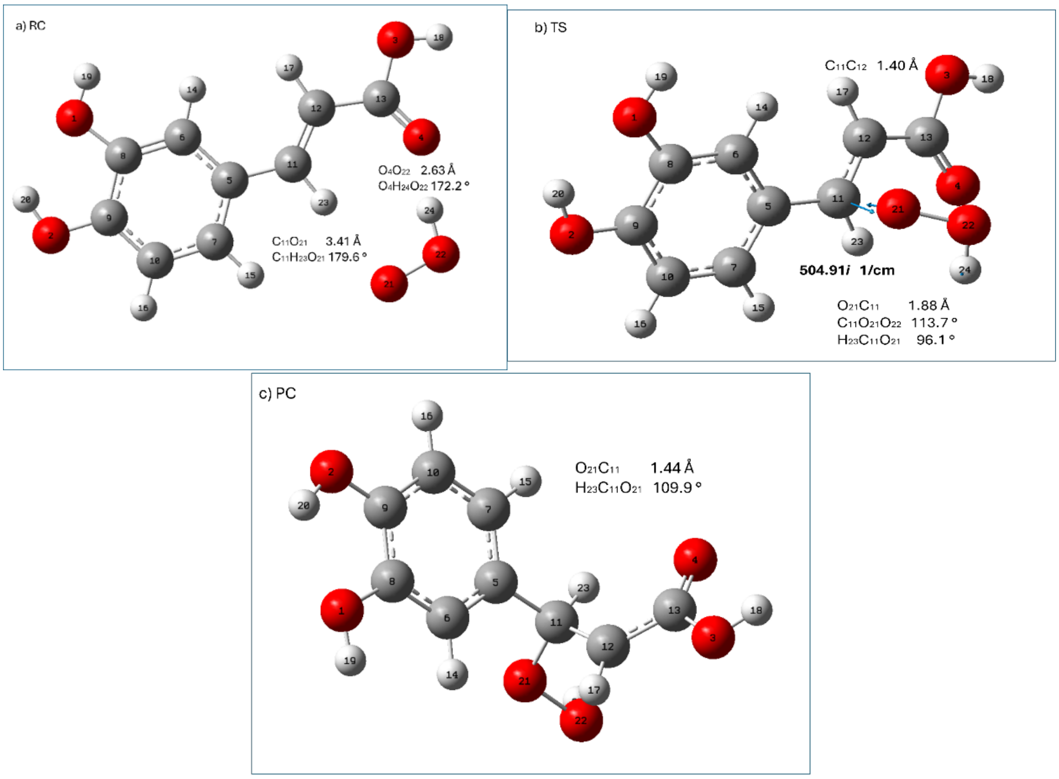

Figure S3. Graphical presentation of the stationary points ((**a**) reactant complex (RC), (**b**) transition state (TS), (**c**) product complex (PC)) encountered along the RAF (C_{11}) reaction pathway of caffeic acid (neutral form) with hydroperoxyl radical. Displacement vectors of imaginary frequency at 504.91*i* are shown as blue arrows.
